# Intraoperative quantitative crystalline lens nuclear opacities analysis based on crystalline lenSx platform

**DOI:** 10.1186/s12886-024-03431-8

**Published:** 2024-05-06

**Authors:** Ying Zhang, Yaya Zhang, Junting Zhang, Tao Wang, Luhui Yi, Yao Zeng, Guorong Zeng, Lingdong Kong, Bo Ye, Yunmin Yi

**Affiliations:** 1 Department of Ophthalmology, Jiangxi Provincial Hospital of Integrated Chinese and Western Medicine, the Fourth Affiliated Hospital of Jiangxi University of Traditional Chinese Medicine, Nanchang, 330003 China; 2 Nanchang Aier Eye Hospital, Nanchang, 330006 China; 3 Affiliated Eye Hospital of Nanchang University, Jiangxi Medical College, Nanchang University, Nanchang, 330006 China; 4 The Fourth Affiliated Hospital, Jiangxi Medical College, Nanchang University, Nanchang, 330006 China

**Keywords:** Femtosecond laser assisted cataract surgery, Spectral domain optical coherence tomography imaging, Lens nucleus density, Clinical prediction model

## Abstract

**Purpose:**

The main objective is to quantify the lens nuclear opacity using spectral-domain optical coherence tomography (SD-OCT) and to evaluate its association with Lens Opacities Classification System III (LOCS-III) system, lens thickness (LT), and surgical parameters. The secondary objective is to assess the diagnostic model performance for hard nuclear cataract.

**Methods:**

This study included 70 eyes of 57 adults with cataract, with 49 (70%) and 21 (30%) in training and validation cohort, respectively. Correlations of the average nuclear density (AND) /maximum nuclear density (MND) with LOCS-III scores, LT, and surgical parameters were analyzed. Univariate and multivariate logistic regression analysis, receiver operating characteristic curves and calibration curves were performed for the diagnostic of hard nuclear cataract.

**Results:**

The pre-operative uncorrected distance visual acuity (UDVA), intraocular pressure (IOP), mean axial length (AL), and LT were 1.20 ± 0.47 log MAR, 15.50 ± 2.87 mmHg, 27.34 ± 3.77 mm and 4.32 ± 0.45 mm, respectively. The average nuclear opalescence (NO) and nuclear colour (NC) scores were 3.61 ± 0.94 and 3.50 ± 0.91 (ranging from 1.00 to 6.90), respectively. The average AND and MND were 137.94 ± 17.01 and 230.01 ± 8.91, respectively. NC and NO scores both significantly correlated with the AND (rNC = 0.733, *p* = 0.000; rNO = 0.755, *p* = 0.000) and MND (rNC = 0.643, *p* = 0.000; rNO = 0.634, *p* = 0.000). In the training cohort, the area under the curve (AUC) of the model was 0.769 (*P* < 0.001, 95%CI 0.620–0.919), which had a good degree of differentiation (Fig. 2a). The calibration curve showed good agreement between predicted and actual probability.

**Conclusion:**

The nuclear density measurement on SD-OCT images can serve as an objective and reliable indicator for quantifying nuclear density.

## Introduction

Cataract is a leading cause of visual impairment worldwide, affecting millions of people each year [1]. The condition is characterized by the clouding of the eye's natural crystalline lens, which can cause blurry vision, sensitivity to light, and other visual disturbances [2]. The escalating prevalence of precataract crystalline lens conditions on a global scale is emerging as a significant focal point within the realm of public health. Cataract surgery is the most common intraocular surgery performed worldwide, with millions of procedures performed each year [3]. Nowadays, cataract surgery has evolved from intracapsular cataract extraction to phacoemulsification. Femtosecond laser-assisted cataract surgery (FLACS) is the latest revolution in the history of cataract. Though the technological advances are undeniable, the cost is high, and most ophthalmologic services still use the phacoemulsification technique for cataract surgery [4].

Classification and quantification of cataract have important clinical applications, including selecting the appropriate surgical technique for cataract surgery, monitoring disease progression, and evaluating the outcomes of cataract surgery. The methods used for grading cataract density can be divided into subjective and objective methods [5]. Subjective methods include the Crystalline lens Opacities Classification System III (LOCS-III), the Wilmer System, the Oxford Clinical Cataract Classification and Grading System, the Wisconsin cataract grading system, and the WHO’s Simplified Cataract Grading System [6–10]. Most grading systems are detailed classification based on standardized images. Nowadays, the LOCS-III, the most widely used system, provides a standardized method for grading cataracts based on their severity and location by comparing the crystalline lens images obtained under a slit lamp with reference crystalline lens images [11]. Although previous studies had shown that LOCS-III has high repeatability, the evaluation differences caused by its subjective grading process cannot be ignored. The grading process is dependent on the experience and training of the graders, which can lead to variability in grading between different graders. Before Femtosecond laser and intraoperative measurements and after LOCS III grading, devices such as the Pentacam(Pentacam nuclear Staging) are used to grade cataracts [12, 13]. Nowdays, cataract researchers have applied computerized image analysis techniques to measure cataract density.

The introduction of femtosecond laser systems has been a significant advancement in cataract surgery. In recent years, FLACS has gained widespread acceptance worldwide. This new technology offers several advantages over traditional techniques, including precise arcuate corneal incisions, adjustable opening diameters of capsular, and crystalline lens fragmentation [14]. The Crystalline lenSx system is the first commercially available FLACS system that utilizes spectral-domain optical coherence tomography (SD-OCT) to localize and measure anterior segment structures, such as the cornea, anterior chamber, and crystalline lens [15]. OCT is a non-invasive, non-contact imaging technique that provides micrometer-level resolution of internal tissue structures using optical coherence [16]. Recently, portable OCT system were designed with Fourier-domain OCT technologies, including SD-OCT and swept-source OCT (SS-OCT) [17]. SD-OCT is the second generation of OCT technology and has significant advantages over traditional first-generation OCT technology in terms of imaging speed, signal-to-noise ratio, and sensitivity [18]. SD-OCT has played an important role in ophthalmology, providing detailed images of the eye's structures and assisting in the diagnosis and management of various eye diseases. However, the current understanding of the applicability of SD-OCT in quantifying nuclear opacities is limited. The objective degree of nuclear opacities measured by SD-OCT during cataract surgery may predict the ultrasound energy and time required for cataract surgery, improve the guidance of surgical plans and parameter settings.

Therefore, this study is aimed to quantify nuclear opacities through SD-OCT and assess the association of the crystalline lens density with LOCS-III scores and phacoemulsification parameters.

## Material and methods

### Patients

This cross-sectional trial included 70 eyes of 65 adults that underwent FLACS surgery at Aier Eye Hospital of Nanchang from September 1, 2022, to May 1, 2023. Participants satisfying any of the following conditions were excluded from the trial: eyes unsuitable for FLACS, such as small pupils (< 6 mm diameter); patient has a history of any kind of ocular trauma or surgery; other eye diseases such as keratoconus, glaucoma, optic atrophy, retinal detachment, etc.; poor image quality caused by poor patient cooperation ability. All patients were randomly divided into model training set (*n* = 49) and model validation set (*n* = 21) in a 7:3 ratio. In this trial, all participants provided informed consent after receiving a thorough explanation of the surgery. This trial was approved by the local ethics committee and followed the tenets of the Declaration of Helsinki.

### Ophthalmic examinations

All participants in this trial underwent a complete ophthalmic examination, including uncorrected distance visual acuity (UCVA), intraocular pressure (IOP), refraction, slit lamp examination under dilated pupils, and a fundus exam. The IOL Master 700 (Zeiss), a non-invasive device that employs advanced technology to capture highly precise measurements of optical biometric data of the eyes, was used to evaluate axial crystalline length (AL) and lens thickness (LT).

Compound tropicamide eye drops (0.5% tropicamide, 0.5% phenylephrine hydrochloride) were used to dilate adequately the patients' pupils. Two experienced cataract surgeons compared the information of crystalline lens morphology observed under the slit lamp with the LOCS-III standard pictures without knowing the specific information of the patient and confirm the crystalline lens opacities grading. The evaluator provided ratings for the level of nuclear opacity (NO) and nuclear color (NC) ranging from 0.1 to 6.9, while assigning scores for cortical opacity and posterior subcapsular opacity on a scale from 0.1 to 5.9. For this investigation, only the NO and NC ratings were utilized to assess connections with SD-OCT measurements [19]. When there was a discrepancy between the two results, a senior chief physician will make the final diagnosis.

### Surgical procedure

All surgeries were performed by the same experienced cataract surgeon, and all data was collected by the same assistant physician. Before ultrasound phacoemulsification and intraocular crystalline len (IOL) implantation, FLACS were performed using Crystalline lenSx system. After application of topical anesthetic with oxybuprocaine hydrochloride 4.0 mg/ml unit dose, the patients' eye was docked to Crystalline lenSx system using a suction device coupled to the laser. SD-OCT incorporated in Crystalline lenSx system provided the anterior segment structures, including the cornea, anterior chamber, and crystalline lens. Corneal incisions, capsulotomy, and crystalline lens fragmentation parameters were visualized by SD-OCT. The surgeon then assessed whether it is necessary to adjust the laser parameters and selected the treatment patterns. The attempted capsulotomy diameter was 5.5 mm in all cases. The crystalline len soften pattern including 3 cross sections with chop diameter of 5.0 mm and 1 central chop cylinder with a diameter of 3.0 mm. The laser pulse energy for crystalline lens fragmentation was 7.00 uJ and 5.00 uJ for capsulotomy.

After the laser treatments were finished, the patient was transferred to another operating room for ultrasound phacoemulsification and intraocular crystalline lens (IOL) implantation. The main incision of 2.4 mm was made on the clear cornea by a 2.4-mm keratome knife. Then, viscoelastic agent was injected into the anterior chamber and the side-port incision was created by a side-port 15-degree blade(Although precise corneal incisions are one of the great advantages of FLACS, experienced doctors are still accustomed to making corneal incisions by hands to shorten the surgical time). The detached anterior capsule was removed with capsule forceps and the direct chop technique was used to perform the phacoemulsification. After phacoemulsification, ZCB intraocular crystalline lens was implanted in the capsular bag through the 2.4 mm main incision. At the end of surgery, PHACO time (s) and CDE (%-s) were recorded on the PHACO machine.

### Crystalline lens density quantification

Before the surgery, mydriatic agent was used to dilate the patients' pupils to obtain a clear cross-sectional SD-OCT image of crystalline lens. The examiner's adjustments to the Crystalline lenSX system during the examination helped to acquire the best quality images. Images captured by SD-OCT during surgery were exported from the Crystalline lenSX system for further analysis. The images with poor quality were discarded.

ImageJ is a Java-based image processing program developed at the National Institutes of Health and the Laboratory for Optical and Computational Instrumentation (LOCI, University of Wisconsin) and is widely used in the field of ophthalmic research [20]. Open the images of crystalline lens in ImageJ software, then convert it to 8-bit grayscale images. The contour of crystalline lens nucleus were outlined manually to provide an integrated gray scale value for further statistical analysis (Intensity pixel value: black-white, 0–255). The average nuclear density (AND) and the maximum nuclear density (MND) are the average and maximum pixel intensities, respectively, of the entire nuclear area.

### Statistical analyses

Data were collected, tabulated, and statistically processed using SPSS version 25.0. Shapiro–Wilk tests were used to verify the normality of quantitative variables. Normally distributed continuous variables were expressed as mean ± standard deviation (SD), and non-normally distributed continuous variables as median (interquartile range). Spearman’s correlation analysis was used to analyze the correlation between quantitative variables. Receiver-operating characteristic (ROC) curves were used with optimal cutoff values determined using Youden’s J index (sensitivity + specificity—1). The logistic analysis was performed to calculate the OR and 95% CI of AND or MND for hard nuclear cataract in training cohort, and a discrimination function was constructed. The area under the curve (AUC), also known as the c-statistic, was used to test model discrimination. The Hosmer–Lemeshow goodness-of-fit test was used to test model calibration.

## Results

Seventy eyes of 57 patients with a mean age of 60.83 ± 12.14 years were included in this study, with 49 (70%) and 21 (30%) in training and validation cohort, respectively. Table 1 shows the baseline demographics and clinical characteristics of the participants. The pre-operative mean uncorrected distance visual acuity (UDVA), intraocular pressure (IOP), mean axial length (AL), and lens thickness (LT) were 1.20 ± 0.47 log MAR (logarithmic minimal angle resolution), 15.50 ± 2.87 mm Hg, 27.34 ± 3.77 mm and 4.32 ± 0.45 mm, respectively. After operation, the UDVA and IOP were 0.39 ± 0.28 log MAR and 15.87 ± 3.31 mm Hg, respectively. At 1 week after operation, the UDVA and IOP were 0.27 ± 0.26 log MAR and 15.57 ± 2.72 mmHg, respectively. No clinical complication was observed.

**Table 1:** Baseline characteristics of the included patients

Characteristics	Total n (%) or m ± sd	Training cohort n (%) or m ± sd	Validation cohort n (%) or m ± sd	*P* value
Eyes	70 (100)	49 (70)	21 (30)	
Female	39 (55.7)	29 (59)	10 (47)	0.543
Age (y)	60.83 ± 12.14	60.81 ± 1.85	60.86 ± 2.28	0.110
High myopia history	27 (100)	15 (49)	12 (21)	0.135
NO	3.61 ± 0.94	3.51 ± 0.92	3.86 ± 0.96	0.153
NC	3.50 ± 0.91	3.42 ± 0.88	3.73 ± 0.96	0.163
UDVA preoperation (logMAR)	1.20 ± 0.47	1.14 ± 0.46	1.34 ± 0.47	0.089
IOP preoperation (mm Hg)	15.50 ± 2.87	15.24 ± 2.75	16.15 ± 3.20	0.235
AL (mm)	27.34 ± 3.77	26.87 ± 3.62	28.54 ± 3.94	0.076
LT (mm)	4.32 ± 0.45	4.31 ± 0.45	4.35 ± 0.45	0.639
AND	137.94 ± 17.01	136.44 ± 17.45	141.70 ± 15.93	0.205
MND	230.01 ± 8.91	230.34 ± 9.11	229.20 ± 8.57	0.906
CDE (%-seconds)	7.08 ± 4.33	6.62 ± 4.25	8.25 ± 4.41	0.164
First-day postoperative UDVA (logMAR)	0.39 ± 0.28	0.33 ± 0.23	0.44 ± 0.32	0.156
First-day postoperative IOP (mm Hg)	15.87 ± 3.31	15.50 ± 3.05	16.80 ± 3.82	0.079
First-week postoperative UDVA (logMAR)	0.27 ± 0.26	0.22 ± 0.21	0.29 ± 0.21	0.075
First-week postoperative IOP (mm Hg)	15.57 ± 2.72	15.46 ± 2.82	15.85 ± 2.50	0.312

*NO* Nuclear opalescence, *NC* Nuclear colour, *UDVA* Uncorrected distance visual acuity, *logMAR* Logarithmic minimal angle resolution, *IOP* Intraocular pressure, *AL* Axial length, *LT* Lens thickness, *AND* Average nuclear density, *MND* Maximum nuclear density, *CDE* Cumulative dissipated energy

**Fig. 1:**
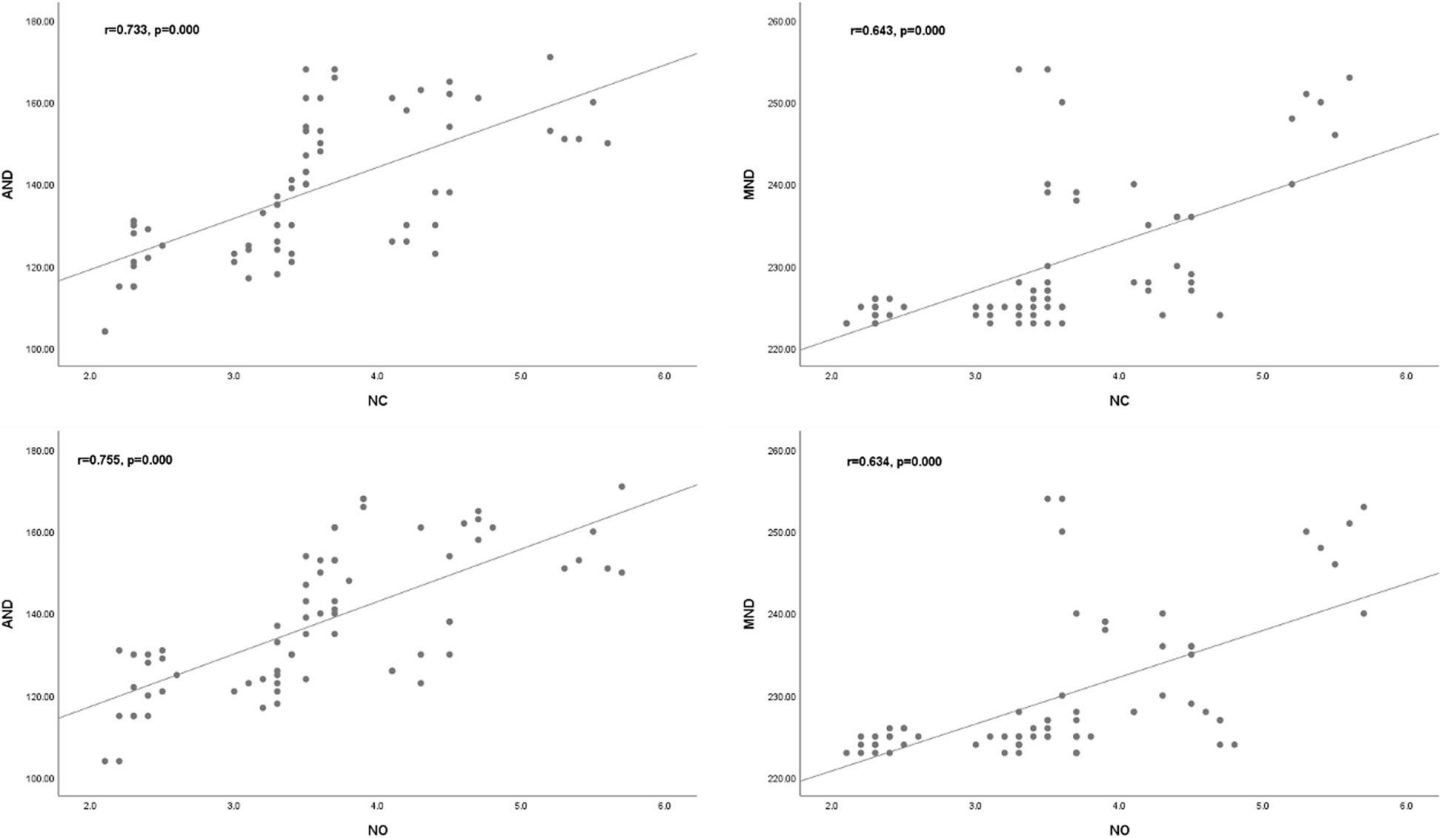
Scatter plots indicate the correlation of nuclear opacity measured by SD-OCT with LOCS-III scores

For the LOCS III grading, the average NO and NC scores were 3.61 ± 0.94 and 3.50 ± 0.91 (ranging from 1.00 to 6.90), respectively. The NO and NC scores distribution did not differ between the training and validation cohort (*p* > 0.05). For SD-OCT-based nuclear opacity quantification, the AND and MND were 137.94 ± 17.01 and 230.01 ± 8.91 (ranging from 0 to 255), respectively. Figure 1 showed the consistency of nuclear opacity measured by SD-OCT and LOCS III scores. NC and NO scores both significantly correlated with the AND (r_NC_ = 0.733, p = 0.000;r_NO_ = 0.755, *p* = 0.000) and MND (r_NC_ = 0.643, *p* = 0.000;r_NO_ = 0.634, *p* = 0.000) from SD-OCT images.

Table 2 indicated the correlations of NO, NC, AND, and MND with visual acuity, CDE, and LT. AND, MND, NO, and NC were significantly associated with the UDVA at baseline and CDE, with the Spearman's p ranging from 0.306 to 0.718 (all *p* < 0.05), but not with LT (all *p* > 0.05). The AND were most correlated with UDVA at baseline and CDE, with the correlation coefficient were 0.349 and 0.718, respectively. What’s more, the LT was weakly correlated with CDE (*r* = 0.226, *p* = 0.040).

**Table 2:** Spearman’s correlation analyses of nuclear density measured by SD-OCT and LOCS-III scores with visual acuity, lens thickness and cumulative dissipated energy in training cohort

	AND		MND		NO		NC	
	R	P	R	P	R	P	R	P
UDVA at baseline	0.349	0.003	0.336	0.005	0.310	0.009	0.306	0.010
First-day postoperative UDVA	0.322	0.007	0.206	0.087	0.126	0.299	0.120	0.321
LT	0.053	0.664	0.297	0.013	0.221	0.066	0.230	0.055
First-week postoperative UDVA	0.147	0.226	0.035	0.774	0.014	0.911	0.001	0.996
CDE	0.718	0.000	0.341	0.004	0.617	0.000	0.623	0.000

*LT* Lens thickness, *CDE* Cumulative dissipated energy, *UDVA* Uncorrected distance visual acuity, *NO* Nuclear opacity, *NC* Nuclear color, *AND* The average nuclear density, *MND* The maximum nuclear density

In the training cohort, variables such as age, gender, high myopia history, AND, and MND, etc. were included in univariate analysis, and finally, the AND variable was included in multivariate logistic regression analysis. The AND index was used to construct a prediction model for the diagnosis of hard nucleus cataract.

**Fig. 2:**
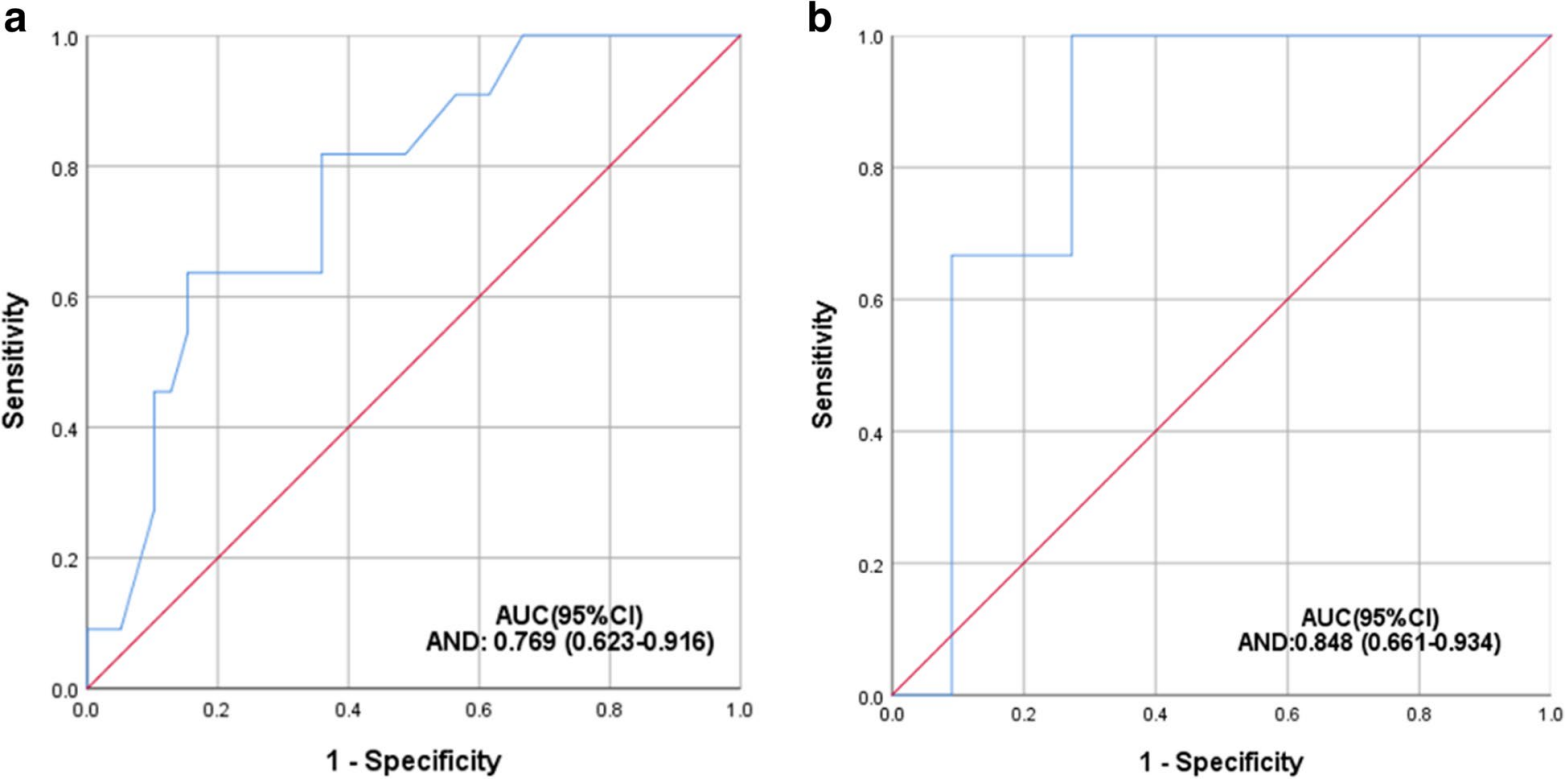
**a** ROC curve of hard nuclear cataract in training cohort. **b** ROC curve of hard nuclear cataract in validation cohort

**Fig. 3:**
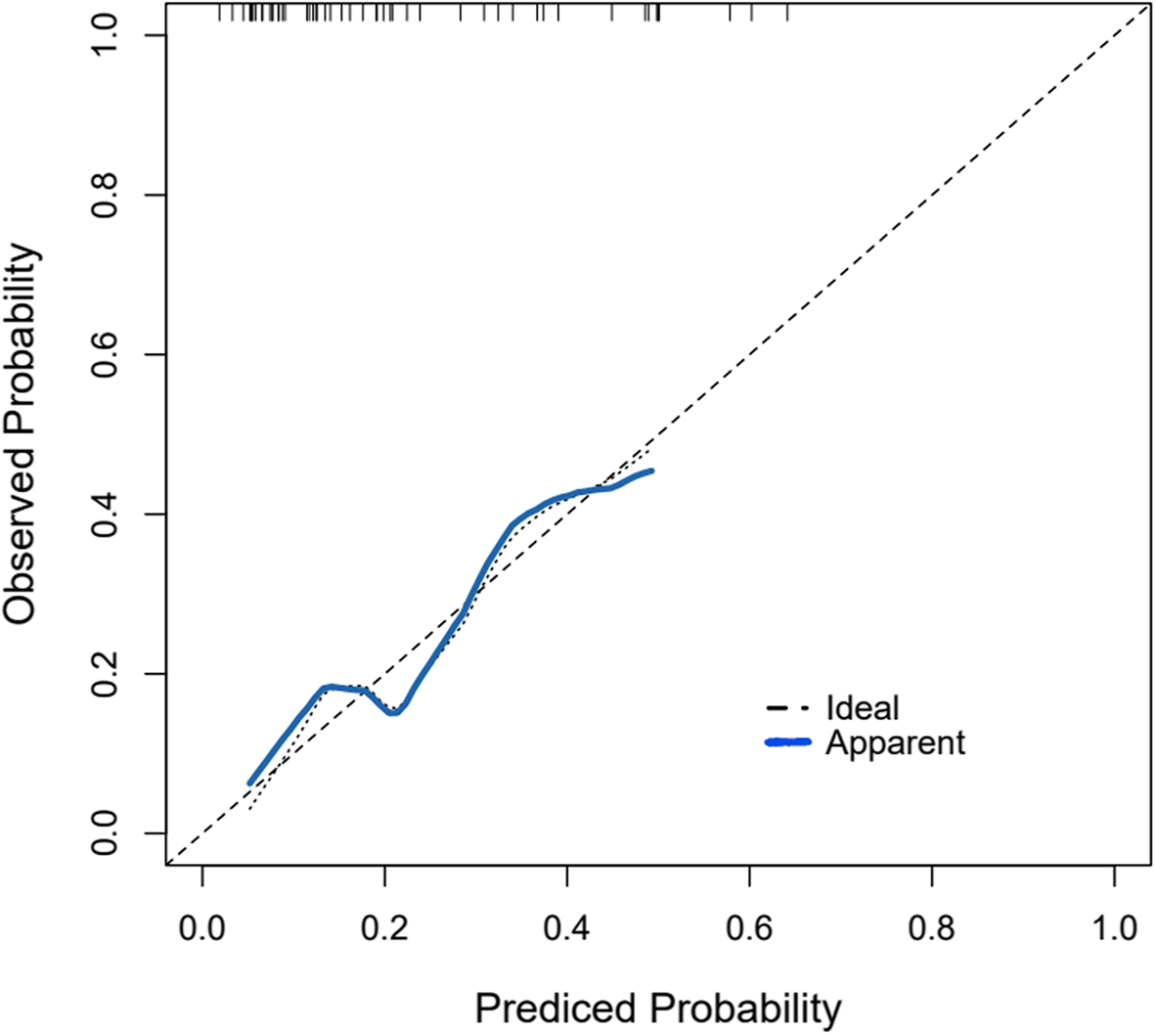
Calibration curve of diagnostic prediction model in training set

In the training cohort, the AUC of the model was 0.769 (*P* < 0.001, 95%CI 0.620–0.919), which had a good degree of differentiation (Fig. 2a). The cut-off value of AND is 150.5, which can distinguish patients with or without hard nucleus cataract, with a sensitivity of 63.6% and a specificity of 84.6%. In the validation cohort, the AUC was 0.848 (*P* = 0.009, 95%CI 0.661–1.000) (Fig. 2b). As is shown in the Table 3, when the diagnostic boundary value of the training cohort was used in the validation cohort, the sensitivity and specificity were 88.9% and 72.7%, respectively. The calibration of the model was evaluated by the Hosmer–Lemeshow goodness-of-fit test. The results showed that Hosmer Limeshow X^2^ = 4.829, *P* = 0.681, indicating that there was no statistically significant difference between the predicted values of the model and the actual observed values. This prediction model has good calibration ability (Fig. 3).

**Table 3:** The Model diagnosis results in the validation cohort

Predictive diagnosis	Pathology diagnosis		Total
	Positive	Negative	
Positive	8	3	16
Negative	1	8	4
Total	9	11	20

## Discussion

In the present study, we evaluated the association of the crystalline lens density with LOCS-III scores and phacoemulsification parameters. A significant, strong correlation was detected between crystalline lens density measured by SD-OCT and LOCS-III scores, phacoemulsification parameters, which indicated its importance in surgical planning and clinical decisions. When AND > 150.5 pixel-units, it is reasonable to suspect hard nucleus cataract, and caution should be exercised when treating cataracts.

In cataract surgery, the majority of the phacoemulsification energy is used to remove the lens nucleus. Therefore, accurate detection of the true density of the lens and preoperative identification of phacoemulsification parameters can provide surgical advantages. However, in many cases, cataract surgeries are initiated using routine parameters, and minor modifications are made during surgery. This can result in the use of excessive phacoemulsification energy in patients with lower lens nucleus density, which can lead to prolongation of phacoemulsification time, total ultrasound time, and increased CDE due to insufficient power in those with high lens nucleus density.

The LOCS III and other subjective methods used to detect and grade nuclear opacities are prone to observer-related errors. This is because the grading is based on the subjective interpretation of the observer, which can vary depending on factors such as lighting conditions, experience, and individual variability. While efforts have been made to standardize grading criteria and reduce observer bias, it is impossible to completely eliminate observer-related errors in subjective grading methods. Therefore, objective methods, such as the Pentacam Scheimpflug system, are becoming increasingly popular for diagnosing and monitoring nuclear cataracts [21, 22]. These systems provide objective measurements that are not subject to observer bias and can help improve the accuracy and reliability of cataract grading.

OCT is a non-invasive imaging technique that uses light waves to create high-resolution images of the eye. The ability to obtain cross-sectional and 3D images with OCT has revolutionized the diagnosis and management of ocular diseases, allowing for earlier detection and more precise monitoring of disease progression. OCT imaging of the anterior segment of the eye has been demonstrated in animal models and in humans using spectral-domain OCT (SD-OCT) and swept-source OCT (SS-OCT) [23, 24]. Our study was consistent with previous studies based on SS-OCT. Wu et al. reported that the association between the CDE and the nuclear density measured by SS-OCT was strong [25]. Chen et al. also found that SS-OCT-based nuclear density was significantly correlated with NO and NC [26]. In this study, we also observed a linear relationship among the CDE, NO, NC and nuclear density measured by SD-OCT. What’s more, we found the CDE had stronger correlations with AND and MND measured by SD-OCT than NO and NC scores. It suggested that AND and MND might be excellent indicators during the FLACS. This allows for more precise and individualized adjustments to the phacoemulsification parameters, which can lead to improved surgical outcomes and reduced complications.

However, attempts at qualitative and quantitative assessment of OCT images of cataract have been limited to the analysis of single cross-sectional images [21, 27]. This is because opacifications in the crystalline lens can appear as multifocal micrometer spots, and some early forms of cataract are located in the lens periphery that develop towards the visual axis. Therefore, 2D imaging of cataract eyes does not explore the full potential of current technology, and it may lead to incorrect diagnosis.

This study also showed that the LT was weakly correlated with CDE (*r* = 0.226, *p* = 0.040). However, Mohamed’s study indicated that laser fragmentation energy and total laser time had a strong positive correlation with LT (*r* = 0.53, *P* < 0 0.001; *r* = 0.31, *P* < 0.001) [28]. We believe that there are two reasons to explain this difference. Firstly, this difference might stem from the different devices used by researchers on both sides to measure LT. Mohamed used SD-OCT to measure LT during surgery, which is based on the principle of optical coherent interference. While we used IOL-Master 700 to measure LT before surgery, which is based on SS-OCT, which is more precise than SD-OCT. There are currently multiple instruments available for in vivo measurement of LT for clinical and scientific research choices. Among these methods, Orbscan II, Pentacam, Lenstar and OCT all can be used to measure LT [24, 29–31]. Secondly, some of our subjects are patients with high myopia and patients with high lens density, which need more comprehensive analysis to balance the difference.

Previous research has already proposed using mean Pentacam nucleus staging and objective scatter index as diagnostic indicators for cataract [32]. In our study, we set 150.5 pixel-units of AND as the cut-off threshold, with a sensitivity of 63.6% and a specificity of 84.6% in the training cohort. When the diagnostic boundary value of the training cohort was used in the validation cohort, the sensitivity and specificity were 88.9% and 72.7%, respectively. The calibration analysis confirmed the good performance. Therefore, using AND can effectively detect hard nuclear cataract. However, this study is limited by its small sample size. In the future, we will continue to expand the sample size to complete further research.

In conclusion, the nuclear density measurement on SD-OCT images can be used as an objective and reliable indicator for quantifying nuclear density. Further technology upgrade should include an intelligent laser platform that adjust laser energy based on the lens nucleus opacity measured by SD-OCT.

### Value statement

#### What was known

- Objective and accurate evaluation of the degree of lens opacity in cataracts is the key to making surgical plans.
- The LOCS-III is the most widely used in clinical for grading cataracts based on their severity and location, but it is a subjective assessment.

### What this paper adds

• The nuclear density was measured by SD-OCT images, which was an objective and reliable indicator for quantifying nuclear density. It can provide the basis for the selection of surgical parameters in the operation.

## Data Availability

The data supporting the results reported in this article are not publicly available but can be accessed by communicating with the corresponding author.
